# Changes in Behaviour and Voluntary Physical Activity Exhibited by Sled Dogs throughout Incremental Exercise Conditioning and Intermittent Rest Days

**DOI:** 10.3390/ani11010118

**Published:** 2021-01-08

**Authors:** Eve Robinson, Emma Thornton, James R. Templeman, Candace C. Croney, Lee Niel, Anna K. Shoveller

**Affiliations:** 1 Department of Animal Biosciences, University of Guelph, Guelph, ON N1G 2W1, Canada; erobin05@uoguelph.ca (E.R.); ethornto@uoguelph.ca (E.T.); jtemplem@uoguelph.ca (J.R.T.); 2 Department of Comparative Pathobiology, Purdue University, West Lafayette, IN 47907, USA; ccroney@purdue.edu; 3 Department of Animal Science, Purdue University, West Lafayette, IN 47907, USA; 4 Department of Population Medicine, University of Guelph, Guelph, ON N1G 2W1, Canada; niell@uoguelph.ca

**Keywords:** working dogs, behaviour, voluntary activity, endurance exercise, recovery

## Abstract

**Simple Summary:**

It is important to understand how typical exercise regimens for sled dogs affect their behaviour. Furthermore, rest and recovery are an equally important part of a conditioning period. Understanding how behaviour and voluntary physical activity change throughout both exercise and rest periods may assist in optimising conditioning to maximise the exercise capacity of dogs, while keeping their well-being in mind. Sled dogs decreased their voluntary activity and locomotive behaviours as they progressed throughout their training but seem to demonstrate a recovery of the reduced voluntary activity when given two consecutive rest days. These findings could be used by mushers and working dog owners to improve the conditioning periods and overall wellbeing of their sled dogs.

**Abstract:**

Participation in repetitive endurance training decreases sled dogs’ voluntary activity and locomotive behaviours; however, the changes in their voluntary physical activity over consecutive rest days has not been examined to assess exercise-recovery. The objective of this study was to examine the changes in behaviour and voluntary activity of sled dogs throughout repetitive incremental conditioning and intermittent rest days. Fourteen dogs (6 males, 8 females; age 3.7 ± 2.7 years; BW 21.5 ± 2.8 kg) underwent 10 weeks of conditioning. Once a week, 5-min video recordings were taken pre- and post-exercise to measure the time spent performing agonistic behaviours, chewing on the gangline, digging, jumping, lunging, posture changing, sitting, standing and lying. Additionally, voluntary physical activity was measured on a day with an exercise bout during baseline, week 4, 5 and 7 and two consecutive rest days during baseline, week 1, 4, 5 and 7. A repeated-measures mixed model was used to analyse data in SAS (v 9.4.). As dogs progressed through their conditioning, the time spent changing posture prior to an exercise bout decreased (*p* < 0.05), suggesting that dogs may reduce their voluntary locomotive behaviours with increasing exercise. Additionally, dogs were more active during the second consecutive rest day than the first (*p* < 0.05), suggesting that rest days may provide a short-term recovery period.

## 1. Introduction

Sled dogs regularly perform repetitive bouts of endurance exercise when training for, and throughout, a racing season. Regular exercise may decrease boredom and provide physical and mental stimulation for dogs, potentially leading to beneficial behavioural changes such as a decrease in destructive or aggressive behaviours [1]. However, excessive repetitive bouts of strenuous aerobic exercise can have deleterious physiological impacts, such as increasing oxidative stress [2,3], muscle damage [4] and promoting gut dysbiosis [5]. In human athletes, these symptoms may also negatively impact mood, contribute to the onset of fatigue and reduce the motivation to exercise [6]. It is likely that these physiological symptoms similarly impact sporting dogs and may manifest as behavioural changes. Prior to an exercise bout, sled dogs are placed in their respective positions on the gangline, which is the line that tethers the dogs to the musher’s sled or ATV. During this period, dogs demonstrate locomotive behaviours such as jumping, lunging forward, digging, postural changes and agonistic behaviours. It has been previously demonstrated that as sled dogs progress through a repetitive, incremental training period, these locomotive behaviours exhibited prior to exercise decrease [7]. It is possible that this behavioural change is partly caused by exercise-induced fatigue or alterations in mood and motivation to exercise. One way to combat any potential negative behavioural and physiological effects of exercise experienced by sled dogs is through the incorporation of consecutive rest days between training bouts.

Rest and recovery are vital parts of the participation in endurance training. Following an exercise bout, there is a need to recover from an increased heart rate, respiratory rate and internal temperature, as demonstrated in working greyhounds [8]. This initial recovery period has been observed to last up to 30 min but may vary depending on the exercise intensity and individual differences [9]. In the prolonged period following an exercise bout, dogs need to recover from potential oxidative stress [2,3] and glycogen depletion [10]. The recovery from these physiological impacts of exercise is important to ensure dogs progress and improve throughout their endurance training [11]. In humans, a combination of excessive exercise and inadequate recovery can lead to a decline in performance, referred to as overtraining [12]. Overtraining in working dogs has not been defined; however, it is likely that the balance between exercise and recovery is highly influential in the success and performance of sled dogs. Therefore, it may be beneficial to define a behavioural indicator of recovery from exercise, to potentially identify a practical and simple visual marker that can be used to ensure sled dogs progress and improve throughout their conditioning period. In mice, voluntary wheel running has been used as a behavioural measure of recovery from forced exercise [13,14,15]. For example, following 150 min of forced exercise, the level of voluntary wheel running returned to pre-exercise measurements in three consecutive rest days [14]. It is possible that a similar indicator, voluntary physical activity, could be used by working dog owners and trainers to assess recovery from exercise. However, the changes in voluntary physical activity over consecutive rest days has not been previously examined in actively training sled dogs.

The objective of this study is to investigate the effects of 10 weeks of incremental endurance exercise on the behaviour and voluntary activity of sled dogs. First, we hypothesised that participation in repetitive training would decrease the time spent performing locomotive behaviours prior to exercise, and increase the sedentary behaviours following exercise, due to the physiological effects of endurance exercise. Second, we hypothesised that voluntary physical activity performed by sled dogs would increase during two consecutive rest days, indicating a potential recovery from endurance exercise.

## 2. Materials and Methods

### 2.1. Animals, Training Regimen and Diet

All procedures were approved by the Animal Care Committee at the University of Guelph (AUP #4008). Fourteen Siberian Huskies, owned by the same client, (5 neutered males, 1 intact male; 8 intact females) with an average age of 3.7 ± 2.7 years and body weight (BW) of 21.5 ± 2.8 kg were used. All dogs were housed, fed and trained at the same offsite facility previously approved by the University of Guelph’s Animal Care Committee (Rajenn Siberian Huskies, Ayr, ON, Canada). Dogs were unexercised prior to the beginning of the study. During the study, dogs were pair or group-housed in free-run, outdoor kennels that ranged in size from 3.5 to 80 m^2^ and contained between 2 and 10 dogs each. Dogs were exercised in a standard gangline formation (refer to Templeman et al. [16] for diagram) four times a week (Mon, Tues, Thurs and Fri), with the distance run each week increasing incrementally, starting at 3 km and reaching 42 km over a 10 week period. This incremental training period was based on previous research on exercising sled dogs [16]. Dogs ran in the same position on the gangline throughout the study. Distances run were recorded daily, and adjustments were made when necessary according to the ambient weather conditions Table 1. When training, dogs pulled an all-terrain vehicle carrying one passenger, weighing a combined total of approximately 350 kg, while maintaining an average speed of approximately 15 km per hour throughout the study period. Exercise bouts began consistently at 08:30 h.

For two weeks prior to the beginning of the study, all dogs were acclimatised to a dry extruded diet that was formulated to meet or exceed all National Research Council (2006) and The Association of American Feed Control Officials (2016) nutrient recommendations for adult dogs at maintenance (Champion Petfoods L.T., Morinville, AB, Canada) and historical feeding data were used to determine the initial food intake. During week 0, dogs were divided into two groups and fed either a dry extruded diet that was formulated to have an insoluble:soluble fibre ratio of 4:1, or a dry extruded diet that was formulated to have a ratio of 3:1 (for details regarding diet, please refer to Thornton et al. [17]). The diet fed had no effect on behaviour or voluntary physical activity throughout the study (*p* > 0.05), therefore the data were pooled to analyse the effects of exercise independently. Dogs were weighed weekly and food intake was adjusted for dogs to maintain baseline BW. Dogs were fed once a day at approximately 15:00 h and were provided ad libitum access to fresh water.

### 2.2. Behavioural Evaluation

All dogs were recorded, using a digital camera (Sony HDR-CX110 HD Handycam, Sony Corp., Tokyo, Japan), while on the gangline for 5 min pre- and post-exercise on d 1 of each week of the study, based on previous research evaluating sled dog behaviour [7]. Once all dogs were placed in their respective positions on the gangline, video recordings were taken to observe the time spent performing agonistic behaviours, chewing on the gangline, digging, jumping, lunging, posture changing, sitting, standing and lying, Table 2. The same behaviours were recorded immediately upon returning from the exercise bout, while all dogs remained on the gangline. All behavioural analysis was completed by a single individual. The overall time spent observing each behaviour (seconds) was determined from each video.

### 2.3. Activity Monitoring

Three-dimensional accelerometers (Fitbarks, Fitbark Inc., Kansas City, MO, USA) were attached to the collars of each dog to record activity during baseline and weeks 1, 4, 5 and 7. These specific weeks were chosen so as not to interfere with additional ongoing data collection. This equipment has previously been used to evaluate sled dog voluntary physical activity [6]. For each of those weeks, activity was evaluated continuously for two rest days (Saturday and Sunday, no training) and on the first active day of the week (Monday, active training). It was not possible to analyse any rest day data from week 1 due to a software malfunction. While activity was being recorded, any periods of human interaction, such as feeding or training, were noted and subsequently removed from the activity count data. For rest days, 10.5 h of data were used from 06:30 h to 15:00 h and 16:30 h to 18:30 h, for a total of 21 h of rest-day data collected each week. For active days, total activity counts were combined from 2 h post-run (dependent on run finishing time) and 1 h post-feeding (16:30 to 17:30 h), for a total of 3 h of active day data collected each week. Data are expressed as activity counts, which represent physical activity as generated by company algorithms (Fitbarks, Fitbark Inc., Kansas City, MO, USA). Similar to a step count generated by a human activity monitor, the voluntary activity counts described herein represent physical activity and a larger voluntary activity count indicates the dog is more active.

### 2.4. Statistical Analysis

The time spent performing a behaviour was converted into percentage of time ((duration of behaviour/duration of recording) × 100) and used for further analysis. Behavioural data and activity counts were analysed with a repeated-measures mixed model using PROC GLIMMIX of SAS (v.9.4; SAS Institute Inc., Cary, NC, USA), with dog as a random effect and week and week × treatment as fixed effects. Day (either Day 1 or Day 2) was also included as a fixed effect when analysing rest day activity. Week was additionally treated as a repeated measure. When the fixed effects were significant, means were separated using Fisher’s Least Squared Difference and significance was declared at *p* < 0.05, and trends at 0.05 ≤ *p* < 0.10. Results are reported as least square means (LSM) ± standard error (SE). For all models, residuals were tested for homogeneity and normality using the Shapiro–Wilk test and plots. Additionally, correlations were performed using PROC CORR of SAS to evaluate the relationship between ambient temperature, run distance, cumulative distance run, week, activity counts and pre- and post-run behaviours.

## 3. Results

Two dogs were removed from the study because of unrelated health issues. During week 2, one female dog was removed (age 5 years, BW of 21.5 kg) and during week 4, one male dog was removed (age 7 years, BW of 26.4 kg). As to include a robust a data set as possible, and since the dogs were removed because of factors unrelated to the study, all data collected up until their respective points of removal are included in this report.

### 3.1. Pre-Run Exercise Behaviour

Baseline data from one female dog (age 2 years, BW of 19.8 kg) and one male dog (age 1 year, BW of 22.8 kg) as well as week 0 data from one female dog (age 2 years, BW of 19.8 kg), were removed because of repeated human interactions which disrupted the behaviour of the dogs. Because of the limited sample size, data from all dogs were used in subsequent weeks, even those whose data were removed from baseline. Week had an effect on the time spent performing agonistic behaviours, changing posture, standing and lying down prior to an exercise bout (*p* < 0.05; Table 3). Dogs spent more time performing agonistic behaviours at baseline than any other week, except week 1 (*p* < 0.05; Table 3). Dogs spent more time changing posture during week 2 than weeks 0 and 3 to 8 (*p* < 0.05; Table 3). Dogs spent more time standing on the gangline in weeks 3 to 8 than during baseline, and more time during week 6 than baseline to week 4 (*p* < 0.05, Table 3). Dogs spent more time lying down during weeks 4 and 5 than at baseline and at weeks 1, 3, 6 and 8 (*p* < 0.05; Table 3). Week had no effect on the time spent chewing the gangline, jumping, lunging, or sitting prior to exercise (*p* > 0.05; Table 3).

The cumulative distance the dogs ran during the week prior to behavioural evaluations was negatively correlated with the time spent performing agonistic behaviours (r = −0.27, *p* < 0.05) and time spent changing posture (r = −0.25, *p* < 0.05). Distance run was positively correlated with the time spent standing (r = 0.28, *p* < 0.05) and tended to be positively correlated with time spent lying (r = 0.16, *p* = 0.067). The cumulative distance the dogs ran the week prior had no correlation with the time spent sitting, lunging, jumping, digging or chewing on the gangline pre-run (*p* > 0.05).

Ambient temperature was negatively correlated with the time spent lying pre-run (r = −0.22, *p* < 0.05), but was not correlated with any other behaviour pre-run (*p* > 0.05).

### 3.2. Post-Run Exercise Behaviour

Week had an effect on the time spent standing and lying down following an exercise bout (*p* < 0.05; Table 4). Dogs spent more time lying down in week 8 than any other week except week 1, and more time lying down during weeks 7 than weeks 0 and 3 (*p* < 0.05; Table 4). Dogs spent more time standing during weeks 0 and 3 than any other week (*p* < 0.05; Table 4). Week had no effect on time spent sitting post exercise (*p* > 0.05; Table 4).

The distance the dogs ran was negatively correlated with the time spent standing (r = −0.29, *p* < 0.05) and positively correlated with the time spent sitting (r = 0.23, *p* < 0.05) and lying down (r = 0.18, *p* < 0.05) post run. Ambient temperature was not correlated with any behaviour post-run (*p* > 0.05).

### 3.3. Rest Day Voluntary Physical Activity

**Figure 1:**
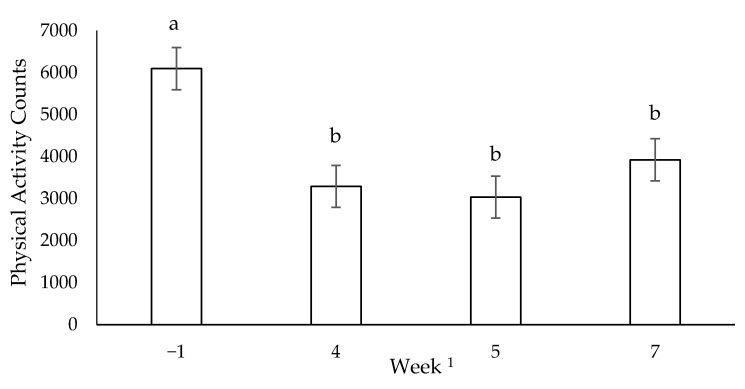
Mean voluntary activity counts during rest days for all dogs (n = 12) throughout 10 weeks of incremental conditioning; ^ab^ columns with different letters are different (*p* < 0.05); error bars represent standard error of the mean; ^1^ distances run were 3 km (week −1), 30.1 km (week 4), 35.8 km (week 5) and 36 km (week 7).

**Figure 2:**
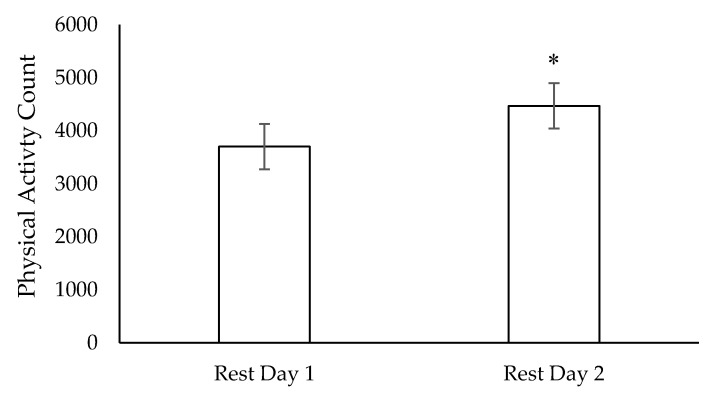
Mean voluntary physical activity counts on first rest day and second rest day of sled dogs (n = 12) undergoing 10 weeks of incremental conditioning; * significantly different (*p* = 0.03); Error bars represent standard error of the mean.

Two dogs (one: 5 years, male, BW of 25.3 kg; and the other: 2 years, female, BW of 19.8 kg) were removed from rest day baseline data. Because of the limited sample size, data from all dogs were used in subsequent weeks, even those whose data were removed from baseline. During both rest days, the dogs’ average voluntary activity was less during weeks 4, 5 and 7 compared to baseline (*p* < 0.05; Figure 1). Rest day activity was negatively correlated to the cumulative distance the dogs ran the previous week (r = −0.52, *p* < 0.05). For all weeks combined, dogs were more active on the second consecutive rest day than on the first rest day (*p* < 0.05; Figure 2).

### 3.4. Run Day Voluntary Physical Activity

**Figure 3:**
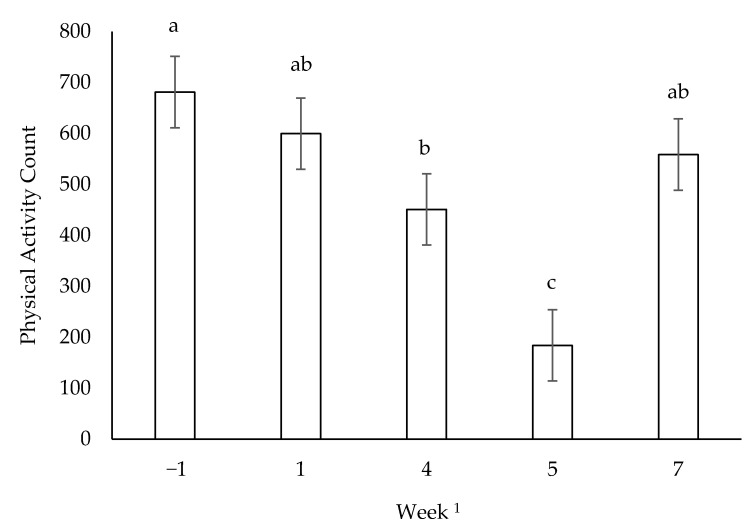
Mean voluntary physical activity counts on run days for all dogs undergoing 10 weeks of incremental conditioning; ^abc^ columns with different letters are different (*p* < 0.05); error bars represent standard error of the mean; n = 14 for weeks −1 and 1; n = 12 for weeks 4, 5 and 7; ^1^ distances run were 3 km (week −1), 11.2 km (week 1), 30.1 km (week 4), 35.8 km (week 5) and 36 km (week 7).

On run days, voluntary activity decreased from baseline to week 5 and increased from week 5 to 7 (*p* < 0.05; Figure 3). Voluntary activity during run days was negatively correlated to the distance the dogs ran that day (r = −0.43, *p* < 0.05) and the cumulative distance run the previous week (r = −0.51, *p* < 0.05). Ambient temperature was not correlated with off day activity (r = 0.07, *p* > 0.05) or run day activity (r = 0.13, *p* > 0.05).

## 4. Discussions

The objective of the study was to investigate the impact of a 10-week incremental exercise period on the behaviour and voluntary activity of sled dogs. The results of this study support previous findings that as sled dogs progress through a conditioning period, pre- and post-exercise behaviours change in response to both week of training and distances they have run [7]. Furthermore, this study was the first to demonstrate the changes in sled dogs’ voluntary physical activity during intermittent rest days.

Our laboratory has previously reported behavioural changes pre- and post- exercise during a 12-week incremental training period [7]. Behavioural changes throughout the first 10 weeks of training were similar in both the present study and the aforementioned study. Specifically, a decrease in posture changing was observed prior to an exercise bout as dogs progressed through their training, as previously reported [7]. Posture changing consists of restlessness and constant changes in state of motion. This behaviour may indicate boredom, discomfort, motivation to exercise or anticipation to exercise; however, no research has previously aimed to characterise this behaviour. Furthermore, a decrease in undesirable behaviours, such as agonistic behaviours, were observed as dogs participated in exercise training. There are a multitude of complex underlying causes of agonistic behaviour, including territory guarding, competition for resources and genetics [18]. Additionally, a survey of dog owners demonstrated that dogs who were never exercised were more likely to exhibit intraspecific aggression [1]. The participation in the current exercise regimen may have alleviated boredom and restlessness, decreasing the agonistic behaviours. On the other hand, the progressive decrease in posture changing as dogs participate in repetitive exercise could indicate a decrease in the motivation to exercise, potentially due to an accumulation of the physiological symptoms of exercise [19]. In humans, excessive exercise, often referred to as overtraining, can lead to lethargy, decreased motivation to exercise and decreased mood [20]. More research should be performed to determine if there are correlations between posture changing and physiological markers of exercise and fatigue to better understand the factors that may influence how much time dogs spend changing postures. It is additionally possible that the reduction in posture changes and agonistic behaviours observed prior to exercise was due to an acclimation to the training routine, resulting in a generally calmer state prior to running. Furthermore, the time the dogs spent lying down during 5 min post-exercise increased as they progressed through their training period and as the distance of the bout increased, both in the present study and in previous research [7]. It is possible that the time spent lying down may correlate with the length of time it takes to recover from an increased heart rate, respiratory rate and internal temperature [8]; however, a longer behavioural observation period, in addition to the use of physiological measurements, is needed to determine this. Nevertheless, these results advance our knowledge of the actual and potential behavioural impacts of repetitive, incremental training regimen on sled dogs.

The changes in voluntary physical activity during two consecutive rest days were assessed throughout the 10 week training regimen. Along with the initial recovery period to return to a resting heart rate, respiratory rate and temperature, prolonged recovery is needed to restore endogenous energy, glycogen repletion [10,21] and recover from oxidative stress [2]. These physiological effects of exercise are associated with fatigue in humans [22,23,24], and voluntary wheel running in mice [14,25], suggesting they may also lead to changes in voluntary physical activity in dogs. Sled dogs performed more voluntary physical activity on their second rest day, suggesting they may be beginning to recover from the physiological impacts of repetitive endurance exercise by this time. Endurance exercise results in a significant glycogen depletion in sled dogs; however, they are capable of attenuating muscle glycogen usage throughout repetitive training bouts [21,26,27,28]. Complete glycogen repletion typically occurs within 24 h following exercise, and consumption of a high carbohydrate diet may accelerate the repletion in humans and dogs [28,29,30]. The recovery of glycogen during the first rest day could contribute to the increase in voluntary activity on the second rest day. In addition to glycogen depletion, endurance exercise increases creatine kinase concentration in sled dogs [2,26], which is a marker of muscle cell damage and contributes to muscle soreness and fatigue. In untrained humans, creatine kinase levels increased after three consecutive bouts of exercise and returned to pre-exercise levels after two days of rest [31]. It is possible that the dogs in the current study had elevated creatine kinase concentrations, causing muscle soreness, fatigue and a reduction in voluntary locomotion. After the first rest day, a decrease in creatine kinase concentrations may further contribute to the increased voluntary physical activity observed in sled dogs. Future research should attempt to correlate various physiological markers, such as glycogen repletion rates and creatine kinase concentrations, with voluntary physical activity, to assess the recovery of sled dogs over multiple rest days. This could allow voluntary activity to be used by mushers and working dog owners as a visual marker of the recovery period of dogs. Ensuring an adequate recovery period between exercise bouts may help improve the physiological adaptations experienced by sled dogs and reduce the possibility of overtraining [7].

Further findings from the current study also indicate the sled dogs may have adapted to the high levels of repetitive exercise performed on a weekly basis. Activity during run days decreased based on both the distance the dogs ran and the week of the training regimen, up until week five. After this week, the weekly training frequency and distance was reduced because of the inclement weather. Even though similar distances were run during weeks five and seven, the reduction in exercise performance between these weeks could have caused the increase in voluntary activity during week seven. This suggests that the sled dogs might have been rapidly adapting to performing at increased durations of exercise bouts, potentially through improved cardiovascular and respiratory function [32] and skeletal muscle capacity [33]. When the training regimen was reduced, sled dogs appeared to compensate for this by performing more voluntary physical activity. This indicates that conditioned sled dogs are motivated to perform at an increased level of exercise and will self-exercise when not provided with sufficient controlled exercise. While we have previously suggested that repetitive endurance exercise could lead to a decrease in motivation to exercise in sled dogs [7], results from the current study indicate these dogs may actually be motivated and capable of adapting to perform high duration repetitive bouts of endurance exercise. Future research should continue to assess the motivation to exercise in working canines in order to inform decisions about exercise requirements for dogs.

## 5. Conclusions

Changes in behaviour and voluntary activity were observed throughout the repetitive conditioning period. A decline in posture changing and agonistic behaviours through participation in exercise could suggest positive outcomes, such as a reduction in boredom and restlessness. Conversely, pre-exercise behavioural changes could suggest a decrease in motivation to exercise. The increase in voluntary activity following one rest day indicates that sled dogs may experience a short-term recovery period. Sled dog owners should therefore consider using multiple rest days between repetitive training bouts to maximise recovery, which may subsequently improve the performance. Additionally, the increase in voluntary physical activity after a reduction in exercise training suggests dogs can become acclimated to performing high duration repetitive exercise. Future research should assess specific behavioural indicators of motivation to determine if sled dogs are continually motivated to perform repetitive endurance exercise. Overall, these results may be used to improve the training regimens that will maximise the recovery, health and performance of sled dogs.

## Figures and Tables

**Table 1:** Cumulative distance the dogs ran during each week, and distance run and daily temperatures when behavioural observations took place.

Week ^1^	Cumulative Weekly Distance (km)	Distance (km)	Temperature (°C)
Baseline (−1)	12	3	−3.15
0	23.2	5.8	1
1	44.8	11.2	5.3
2	67.8	17.2	−3.8
3	95.2	23.8	5
4	120.4	30.1	−6
5	101.7	35.8	−2
6	81.5	16.2	4
7	108	36	−2
8	148.4	41.4	0

^1^ During baseline (week −1), all dogs were consuming the same diet. Dogs were switched to their allocated diet, containing an insoluble: soluble ratio of 4:1 (control) or 3:1 (treatment), starting at week 0.

**Table 2:** Description of behavioural parameters analysed during 5 min of video taken immediately pre- and post-exercise for actively training sled dogs ^1^.

Behaviour	Description
Agonistic Behaviour	Behaviours associated with social conflict, including noncontact (baring teeth, snapping) and contact (biting, nosing, wrestling)
Chewing	Placing any part of gangline in mouth and performing chewing action
Digging *	Using two front paws to dig at the ground
Jumping *	Upward motion where all four paws leave the ground
Lunging *	Upward and forward motion where front two paws leave the ground
Lying	Positioned with ventral or side body in contact with the ground
Posture Changes *	Frequent changes in state of motion, repeatedly lifting paws, pacing (>3 s)
Sitting	Positioned with rear end and two front paws in contact with the ground
Standing	Upright position with three or four paws in contact with the ground

^1^ Adapted from Robinson et al. [7] * Behaviours classified as locomotive behaviours.

**Table 3:** Percent of time (%) spent performing observed behaviours on the gangline during the 5 min pre-exercise period for dogs undergoing 10 weeks of incremental conditioning.

Behaviour	Week										SEM ^1^	*p*-Value
	−1	0	1	2	3	4	5	6	7	8		
Agonistic	5.4 ^a^	1.5 ^b^	2.7 ^ab^	0.4 ^b^	0.8 ^b^	0 ^b^	0 ^b^	0 ^b^	1.5 ^b^	0 ^b^	1.2	*p* < 0.05
Chewing	0.6	2.0	0.7	1.3	0.2	0.7	0.8	0	0	0.4	0.6	*p* > 0.05
Digging	1.8	0.3	2.3	0.2	0.7	0.5	0.8	0.5	0.4	0.4	1.1	*p* > 0.05
Jumping	0.6	0	0.8	0.3	0	0.1	0	0	0.1	0.3	0.3	*p* > 0.05
Lunging	4.2	0.9	3.8	2.6	2.3	1	1.6	0.3	1.6	3	1.4	*p* > 0.05
Lying	0.8 ^bc^	1.6 ^abc^	0^c^	1.2 ^bc^	0.2 ^c^	4.53 ^a^	4.5 ^ab^	0.1^c^	1.0 ^bc^	0.1^c^	1.4	*p* < 0.05
Postural Changes	32.4 ^bc^	20.1 ^def^	49.8 ^a^	35.9 ^b^	25.9 ^cd^	15.5 ^ef^	16.4 ^ef^	12.1^f^	21.1 ^cde^	21 ^cdef^	5.5	*p* < 0.01
Sitting	5.5	8.4	3	2.3	1.8	6.6	2.4	1.1	0.4	0	2.7	*p* > 0.05
Standing	51.5 ^d^	65.2 ^bc^	36.9 ^e^	54.8 ^cd^	67.5 ^b^	69.9 ^b^	72.8 ^b^	84.7 ^a^	72.9 ^b^	71.9 ^b^	6.8	*p* < 0.01

^1^ Standard error of the mean; n = 14 for weeks 1 and 2; n = 13 for weeks 0, 3 and 4; n = 12 for weeks −1 and weeks 5 to 8. ^abcdef^ Values in a row with a different superscript are different (*p* < 0.05).

**Table 4:** Percent of time (%) spent performing observed behaviours on gangline during the 5-min post-exercise period for dogs undergoing 10 weeks of incremental conditioning.

Behaviour	Week (Distance Ran)										SEM ^1^	*p*-Value
	−1	0	1	2	3	4	5	6	7	8		
	(3 km)	(5.8 km)	(11.2 km)	(17.2 km)	(23.8 km)	(30.1 km)	(35.8 km)	(16.2 km)	(36 km)	(41.1 km)		
Lying	25.9 ^bc^	3.4 ^e^	37.6 ^ab^	27.0 ^bc^	4.4 ^de^	20.1 ^bcde^	12.9 ^bcd^	13.9 ^cde^	32.1 ^b^	52.0 ^a^	8.3	<0.01
Sitting	0	3	1.8	2.9	0.8	9.4	4.6	7.2	11.6	11.1	3.9	>0.05
Standing	74.3 ^bc^	93.5 ^a^	61.6 ^bc^	70.0 ^bc^	94.4 ^a^	70.3 ^bc^	67.5 ^bc^	76.1 ^ab^	54.9 ^c^	35.7 ^d^	8.6	<0.01

^1^ Standard error of the mean; n = 14 for weeks −1 to 2; n = 13 for weeks 3 and 4; n = 12 for weeks 5 to 8; ^abcde^ Values in a row with a different superscript are different (*p* < 0.05).

## Data Availability

The data presented in this study are available on request from the corresponding author.
